# A novel approach for fast detection of sepsis with Gram‐negative bacterial infection

**DOI:** 10.1111/1751-7915.13314

**Published:** 2018-10-22

**Authors:** Jingan Lin, Long Chen, Jiansen Lin, Haiping Wu, Paul Okunieff, Bing Wu, Bing Yang, Jinrong Chen, Jianhua Lin, Lurong Zhang

**Affiliations:** ^1^ First Affiliated Hospital of Fujian Medical University Fuzhou 350005 China; ^2^ Fujian key Lab of Individualized Active Immunotherapy Fuzhou 350005 China; ^3^ Key Lab of Radiation Biology of Fujian Province Universities Fuzhou 350005 China; ^4^ Xiamen Bioendo Technology Co., Ltd. Xiamen 361002 China; ^5^ Department of Radiation Oncology College of Medicine University of Florida Gainesville FL 32610 USA

## Abstract

Sepsis, a life‐threatening systemic infection, requires quick treatment. Gram‐negative bacteria (GNB) are the major causative pathogens and their endotoxin can be a surrogate biomarker for diagnosis. We explored a fast identification of GNB by first culturing blood to increase endotoxin levels and then detecting endotoxin by *Tachypleus* amebocyte lysate (TAL) with kinetic turbidimetric assay (KT‐TAL). Heating samples could significantly increase the endotoxin released from GNB; speed and time of centrifugation, and sample dilution could affect the endotoxin results. At a high GNB load, endotoxin was detected 3 h after culture, 6.5 h earlier than the BD BACTEC blood culture system detecting GNB. At a low GNB load, endotoxin was detected at 9 h after culture, 13 h earlier than by the BD BACTEC system. In a sepsis patient with *Acinetobacter baumannii,* we detected endotoxin at 12 h after culture, while the BD BACTEC system needed 28.5 h for detection, allowing physicians an earlier decision on appropriate treatment.

## Introduction

Sepsis requires immediately appropriative treatment, since each 1‐h delay in diagnosis will increase mortality rates by 5–10% (Pfafflin and Schleicher, 2009; Schuts *et al*., 2016). A fast detection of a blood‐borne pathogen is thus critical for rescuing life (Kibe *et al*., 2011; Turner *et al*., 2017).

At present, blood culture is the gold standard for the diagnosis of sepsis, but it needs blood culture for about 16–28 h (Sarkar *et al*., 2015), hampering early treatment decision. In addition, not all GNB in blood could be detected by the BD BACTEC system (Thomas *et al*., 1984).

Amebocyte Lysate (AL) is the most sensitive reagent to detect the endotoxin of GNB (Cooper *et al*., 1971; Levin *et al*., 1972). The enzymatic clotting cascade can be kinetically recorded with a turbidimetric reader (Jorgensen *et al*., 1973). This sensitive assay has been widely used in detecting the endotoxin in different biosamples (Cooper *et al*., 1972; Jorgensen and Jones, 1975). Compared to the conventional culture assay for GNB, the endotoxin AL assay is more sensitive and reliable (McCartney *et al*., 1983). However, components of plasma/serum can interfere with endotoxin detection at the onset of sepsis. (Gnauck *et al*., 2015; Bottiroli et al., 2017). Hence, it is urgently needed to develop a better approach for detecting endotoxin.

**Figure 1:**
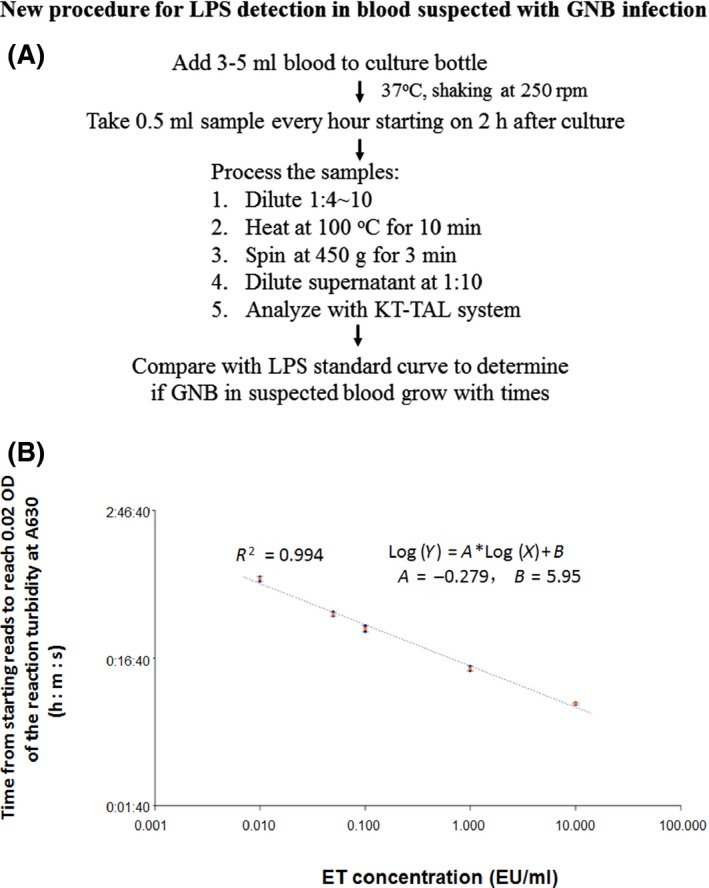
Flow chart for new procedure of LPS detection in blood suspected with GNB infection and its reference standard: (A) 3–5 ml blood was added into culture bottle. 2 h later, 0.5 ml sample was taken every other hour for dynamic test of LPS. The samples were diluted, heated, spun and diluted again before LPS quantitative measurement with KT‐TAL system. (B) The results were compared with LPS standard curve to determine if LPS increased as GNB in suspected blood grew with times. The lyophilized endotoxin standard stock was reconstituted with endotoxin‐free water, diluted to final concentrations of 50, 10, 1, 0.1, 0.01 and 0 EU ml^−1^ on a microplate in triplicates. After added 100 μl TAL reagents into each well, the plate was placed into the kinetic incubating reader (BioTek™ ELx808IULALXH), and the reader immediately started to measure the endotoxin level with the kinetic assay program. The gel formation was recorded every 30 seconds for 30‐60 minutes at wavelength of 630 nm. For this set of study, the formula generated was Log(Y) = A*Log(X) + B, where Y = reaction time (onset time), A = the Y‐intercept, X = endotoxin concentration, B = slope of the regression curve. In this set of study, A was −0.279, B was 5.95, and *R* ^2^ (correlation efficiency) was 0.994. If the standard curve is done with the same batch reagents and the same procedure, then it can be stored in the reader as reference for the following analysis. However, if the reagents change or procedures alter, the new standard needs to be re‐created as a new reference.

We propose the TAL‐based approach shown in Fig. 1 for an early diagnosis and treatment monitoring of GNB sepsis.

In the BD BACTEC system blood, bacteria are cultured and their metabolic products are detected with a fluorescent reporter. We also first cultured the blood bacteria for 1–2 h; 0.5 ml media was collected at 1–2 h intervals and assessed for endotoxin with KT‐TAL assay until detected (Fig. 1A). TAL was added to the sample well, the first OD_630_ reading was taken within 30 s as the background reading,then continued every 30 s until it reached 0.02 (corresponding to an endotoxin level > 14 EU ml^−1^). A < 0.007 EU ml^−1^ reading was regarded as endotoxin negative, and 0.01 EU ml^−1^ was considered as suspected endotoxin positive. If ET level increased in next 1–2 h sample, GNB growth in the test sample was confirmed. An ET standard curve was set up for quantitative measurement of ET (Fig. 1B). The more GNB was in blood, the faster endotoxin could be detected.

Endotoxin exists as free endotoxin and endotoxin associated with intact cell walls (Jorgensen *et al*., 1973). Heating not only promotes the release of endotoxin, but also denatures and precipitates interfering material (Hurley, 1995). Indeed, heating increased the amount of detectable endotoxin (Table 1), thereby shortening the time for endotoxin detection. No increase over time was seen for ET with a culture from a Gram‐positive bacterium (Table 1).

**Table 1:** Effect of heating on ET released from GNB samples and assay specificity.^a^

Bacterial samples	Sampled after hours of culture	ET detected (EU ml^−1^)	
		Unheated	Heated
*E. coli*	4	0.144	6.689*
	5	0.326	10.854*
	6	0.999	> 14.125*
	7	4.237	> 14.125*
	8	10.706	> 14.125*
	9	> 14.125	> 14.125
*Staphylococcus aureus*	4–9	< 0.007	< 0.007

**a.** Sample diluted at 1:4 with ET‐free water, heated at 100°C for 10 min, spin at 450 g for 3 min, and then the supernatant was further diluted at 1:10 for ET measurement with KT‐TAL system. The experiments were repeated three times with the same tendency, that is in the GNB *E. coli* group the ET of heated samples was much higher than the unheated samples (*P <* *0.05), while in Gram‐positive *Staphylococcus aureus* group, there was no difference of ET between the heated and unheated samples.

Since the KT‐TAL assay measures gel formation with endotoxin, it requires a clean and transparent sample. To remove cloudy materials, samples were centrifuged. Centrifugation at 5000 *g* for 3 min leads to a 15% loss of endotoxin, while at 450 *g* for 3 min, only 9% was lost. If the sample is clear and transparent, no centrifugation is necessary.

To define an optimal dilution factor, we took blood samples cultured in BD BACTEC aerobic bottles spiked with 1 EU ml^−1^ of endotoxin. At 1:10 and 1:20 dilution, endotoxin was not or only inefficiently detected, while at 1:40 and 1:100 dilution, endotoxin level close to the spiked 1 EU ml^−1^ endotoxin was recovered (>97%) (Figure S1).

To test if the combination of blood culture with KT‐TAL assay could detect ET of GNB earlier than the conventional BD BACTEC system, the same amounts (0.5 McF) of *E. coli* or *Klebsiella pneumoniae* were diluted at 1:10^−8^ and resulted in a positive reaction after 3–4 h in the KT‐TAL assay compared to 9.5 and 12.5 h in the BD BACTEC system. At the 1:10^−9^ dilutions, the detection time was for *E. coli* 9 and 22 h for KT‐TAL assay and BD BACTEC respectively.

Finally, a patient with suspected and later proven (VITEK 2, bioMérieux) *Acinetobacter baumannii* sepsis was tested. The BD BACTEC system reported pathogen positive with GNB at 28.5 h after culture, while KT‐TAL system detected endotoxin at 12 h, 16.5 h earlier than the conventional BD BACTEC system did.

The TAL‐based turbidimetric assay provides quantitative information, allowing a follow‐up of GNB infection before and after treatment within 1 h assay time that only needs a 37°C shaker for bacteria culture, a microplate reader and TAL reagent kit. However, sepsis with Gram‐positive pathogens will be missed.

## Conflict of interest

None declared. All the blood sample collection was approved by the institutional review board committee of the First Affiliated Hospital at Fujian Medical University (#2013‐29). The operation processes were followed the institutional safety guideline for laboratory involved in handling of pathogen bacteria.

## Supporting information


**Table S1** Optimizations of sample dilution by recovery test of spiked samples.
