# Supplementary material for: A novel approach for fast detection of sepsis with Gram‐negative bacterial infection
Source: Microb Biotechnol. 2018 Oct 22;11(6):1121–3. doi: 10.1111/1751-7915.13314 (PMC6196396; doi:10.1111/1751-7915.13314)
Supplement: Supplementary file 1 — Table S1 Optimizations of sample dilution by recovery test of spiked samples. [file MBT2-11-1121-s001.docx]

| **Bacterial samples** | **Sampled after hours of culture** | **ET detected**  **(EU/ml)**  **No-heated heated** |
| --- | --- | --- |
| *E. coli* | 4 | 0.144 6.689* |
|  | 5 | 0.326 10.854* |
|  | 6 | 0.999 >14.125* |
|  | 7 | 4.237 >14.125* |
|  | 8 | 10.706 >14.125* |
|  | 9 | >14.125 >14.125 |
|  |  |  |
| *Staphylococcus aureus* | 4--9 | <0.007 <0.007 |
|  |  |  |

**s-Table 1. Optimizations of sample dilution by recovery test of spiked samples #**

|  | **M1*** | **M1-S*** | **M1** | **M1-S** | **M1** | | **M1-S** | | **M1** | **M1-S** |
| --- | --- | --- | --- | --- | --- | --- | --- | --- | --- | --- |
| Dilution | **1:10** | | **1:20** | | **1:40** | | | | **1:100** | |
| ET detected (EU/ml) | <0.007 | <0.007 | <0.007 | 0.028 | <0.007 | | | 1.105 | <0.007 | 1.181 |
| ET recovered% | -- | | 2.68% | | 105.69% | | | | 113.01% | |
|  | | | | | | | | | | |
|  | **CN1*** | **CN1-S*** | **CN1** | **CN1-S** | | **CN1** | | **CN1-S** | **CN1** | **CN1-S** |
| Dilution | **1:10** | | **1:20** | | | **1:40** | | | **1:100** | |
| ET detected (EU/ml) | <0.007 | <0.007 | <0.007 | 0.025 | | <0.007 | | 1.244 | <0.007 | 1.064 |
| ET recovered% | -- | | 2.39% | | | 118.99% | | | 101.77% | |
|  | | | | | | | | | | |
|  | **CN2*** | **CN2-S*** | **CN2** | **CN2-S** | | **CN2** | | **CN2-S** | **CN2** | **CN2-S** |
| Dilution | **1:10** | | **1:20** | | | **1:40** | | | **1:100** | |
| ET detected (EU/ml) | <0.007 | <0.007 | <0.007 | 0.01 | | <0.007 | | 1.153 | <0.007 | 1.057 |
| ET recovered% | -- | | 0.96% | | | 110.28% | | | 101.10% | |
|  | | | | | | | | | | |
|  | **CN3*** | **CN3-S*** | **CN3** | **CN3-S** | | **CN3** | | **CN3-S** | **CN3** | **CN3-S** |
| Dilution | **1:10** | | **1:20** | | | **1:40** | | | **1:100** | |
| ET detected (EU/ml) | <0.007 | <0.007 | <0.007 | 0.022 | | <0.007 | | 1.021 | <0.007 | 1.132 |
| ET recovered% | -- | | 2.10% | | | 97.66% | | | 108.27% | |

#: 1 EU/ml of ET was added to all tubes marked with –S, the ET detected should be ~ 1, < 1 EU/ml indicating there were some strong factor inhibiting the assay system.

M1*: culture media plus 3 ml blood as background control.

M1-S*: ET (1 EU/ml) spiked into M1* for ET recovery (%) at different dilutions.

CN1*, CN2*, CN3*: clinical blood culture negative sample from BD bottle without addition of ET as background control.

CN1-S*, CN2-S*, CN3-S*: ET (1 EU/ml) spiked into CN1*, CN2*, CN3* for calculation of ET recovery (%) at different dilutions.
